# Bio‐Inspired Leaf‐Mimicking Nanosheet/Nanotube Heterostructure as a Highly Efficient Oxygen Evolution Catalyst

**DOI:** 10.1002/advs.201500003

**Published:** 2015-03-10

**Authors:** Yongcheng Wang, Kun Jiang, Hui Zhang, Tong Zhou, Jiwei Wang, Wei Wei, Zhongqin Yang, Xuhui Sun, Wen‐Bin Cai, Gengfeng Zheng

**Affiliations:** ^1^ Department of ChemistryLaboratory of Advanced MaterialsCollaborative Innovation Center for Energy MaterialsFudan UniversityShanghaiP.R. China; ^2^ Soochow University‐Western University Centre for Synchrotron Radiation ResearchInstitute of Functional Nano and Soft Materials Laboratory (FUNSOM)Collaborative Innovation Center of Suzhou Nano Science and TechnologySoochow UniversitySuzhouP.R. China; ^3^ State Key Laboratory of Surface PhysicsKey Laboratory for Computational Physical Sciences (MOE) and Department of PhysicsFudan UniversityShanghaiP.R. China

**Keywords:** CoO*_x_*, electrocatalyst, nanosheet, nanotube, oxygen evolution reaction

## Abstract

Plant leaves represent a unique 2D/1D heterostructure for enhanced surface reaction and efficient mass transport. Inspired by plant leaves, a 2D/1D CoO*_x_* heterostructure is developed that is composed of ultrathin CoO*_x_* nanosheets further assembled into a nanotube structure. This bio‐inspired architecture allows a highly active Co^2+^ electronic structure for an efficient oxygen evolution reaction (OER) at the atomic scale, ultrahigh surface area (371 m^2^ g^−1^) for interfacial electrochemical reaction at the nanoscale, and enhanced transport of charge and electrolyte over CoO*_x_* nanotube building blocks at the microscale. Consequently, this CoO*_x_* nanosheet/nanotube heterostructure demonstrates a record‐high OER performance based on cobalt compounds reported so far, with an onset potential of ≈1.46 V versus reversible hydrogen electrode (RHE), a current density of 51.2 mA cm^−2^ at 1.65 V versus RHE, and a Tafel slope of 75 mV dec^−1^. Using the CoO*_x_* nanosheet/nanotube catalyst and a Pt‐mesh, a full water splitting cell with a 1.5‐V battery is also demonstrated.

## 1 Introduction

Electrochemical or photoelectrochemical water splitting represents one of the most promising methods to store electrical or photo energy in chemical bonds, with minimum carbon footprint in the environment.^1^, ^2^, ^3^, ^4^ One of the most critical steps of water splitting is the oxygen evolution reaction (OER), 4 OH^−^ + energy → O_2_ + 2 H_2_O + 4 e^−^, which is a multistep four‐electron process and thus kinetically sluggish.^5^ Iridium and ruthenium oxides (IrO*_x_*, RuO*_x_*) are regarded as pioneering OER catalysts with high efficiencies,^6^ while their prospect of large‐scale application is overwhelmed by their scarcity and the associated high cost. Substantial research efforts have recently been focused on developing new active OER catalysts with earth‐abundant elements.^7^, ^8^ Among them, the cobalt (Co)‐based compounds, including oxides,^9^, ^10^, ^11^ multimetal oxides,^12^, ^13^, ^14^ hydro(oxy)oxides,^15^ phosphates,^16^, ^17^ chalcogenides^18^, ^19^, ^20^ and molecular catalysts,^7^ have been demonstrated as promising substitutes as OER catalysts for noble metal compounds, due to their high activity, stability, environmental benignity, and abundance.

Previous research efforts in design and optimization of Co‐based OER catalysts can be generally categorized into three scale levels. First, at the atomic scale, the tuning of cobalt oxidation state,^21^ coordination,^7^, ^16^ doping,^22^ and composition of complex compounds,^23^, ^24^ allows for the electronic structure optimization for enhancing the four‐electron OER process. Second, at the nano/mesoscale, the design and synthesis of various nanostructures, such as nanoparticles,^9^ nanowires,^22^ nanosheets,^11^ nanotubes^25^ and mesopores,^26^ can provide large surface area, optimized site accessibility, and exposure of active lattice planes for the interfacial electrochemical reactions and enhanced OER activity, compared to bulk Co_3_O_4_ counterparts. Third, at the microscale, the assembly of individual building blocks into an interlacing, conducting and highly porous architecture can benefit efficient mass transport of electrolytes, high electric conductivity and structural stability.^27^, ^28^ Nonetheless, due to the challenges of simultaneous structure control over all these three scale levels, the present OER performances of cobalt compounds are still not comparable to noble metal compounds for practical energy applications.

**Figure 1:**
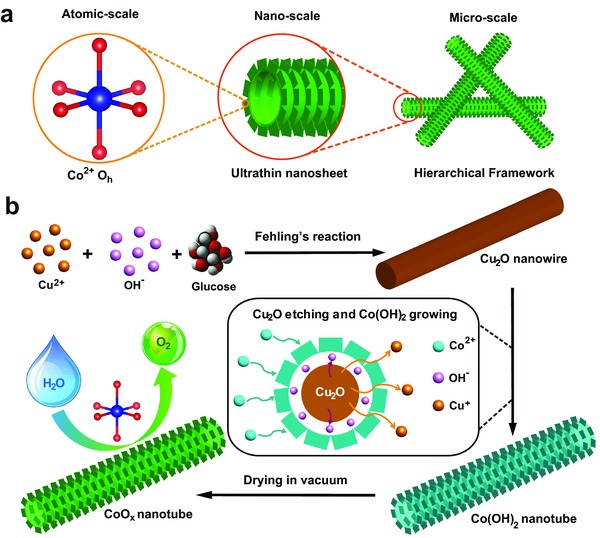
a) Schematic illustration of design of the hierarchical CoO*_x_* nanosheet/nanotube structures with multiple scale optimizations. b) Synthesis procedure of the hierarchical CoO*_x_* nanosheet/nanotube structures, including the in situ growth of Cu_2_O nanowire templates, etching of the nanowire templates and regrowth of ultrathin Co(OH)_2_ nanosheets from the original nanowire template surface, and dehydration to form CoO*_x_* nanosheet/nanotube structure.

As a classical example of nature evolution, the plant leaves represent a unique heterostructure beneficial for their biofunctions. Typically, each leaf is grown into a thin 2D morphology with optimized surface area and reduced overpacking between their neighbors, which favors for enhanced light absorption and surface reactions. Meanwhile, the existence of 1D, hollow tubular structures under the leaf arrays allows for efficient transport of water and nutrition towards the leaves for their reactions and biofunctions. Inspired by the plant leaf structure, herein, we demonstrate the realization of a CoO*_x_* 2D/1D heterostructure, composed of ultrathin CoO*_x_* nanosheets that are further assembled into a nanotube structure, which enables simultaneous structural optimization over different length scales for superior OER activity (**Figure** **1**a). First, the obtained CoO*_x_* has Co^2+^ in octahedral symmetry (O_h_), providing an excellent electronic structure for the OER reaction.^16^ Second, the direct conversion and growth of CoO*_x_* nanosheets over the Cu_2_O nanowire template surface precludes the homogeneous nucleation of CoO*_x_* in solution as well as the accumulation of nanosheet thickness, thus resulting in an ultrathin nanosheet assembly with high density and large surface area. Moreover, these few‐layered CoO*_x_* nanosheets are assembled into nanotubes of tens of micrometer long, which are further interlaced into a large 3D, porous framework for efficient charge and ion transport. This CoO*_x_* nanosheet/nanotube heterostructure has an ultrahigh surface area of 371 m^2^ g^−1^ ever obtained for CoO*_x_*, and, demonstrates the best OER performance based on cobalt compounds reported so far. A low onset potential of ≈1.46 V versus reversible hydrogen electrode (RHE) has been obtained, substantially better than most of the previously reported OER catalysts, with a record‐high current density of 51.2 mA cm^−2^ at 1.65 V versus RHE. Furthermore, using a single 1.5‐V AAA battery as input, a full water splitting cell that couples the CoO*_x_* OER catalyst and a Pt‐mesh hydrogen evolution reaction (HER) catalyst has been demonstrated.

## 2 Result and Discussion

The hierarchical CoO*_x_* nanosheet/nanotube structure is synthesized by a two‐step solution approach, in which Cu_2_O nanowire templates are first obtained via a solution‐based 1D crystal growth method, followed by in situ etching of Cu_2_O nanowire core and regrowth of Co(OH)_2_ nanosheets over the nanowire template surface (Figure 1b). Specifically, Cu_2_O nanowires are synthesized via a Fehling's test reaction with a dislocation‐driven growth mechanism,^29^ in which Cu^2+^ and OH^−^ are used as reactants, and glucose and sodium tartrate are used as the reducing and the coordination reagents, respectively. The existence of tartrate forms a Cu^2+^−tartrate coordination complex (*K*_f_ ≈ 10^7^) that both prevents Cu(OH)_2_ precipitation and reduce the free Cu^2+^ concentration, thus lowering the supersaturation and promoting the dislocation‐driven growth of nanowires. Afterwards, Co(OH)_2_ nanotubes are formed by using a coordination etching and precipitation method,^30^ in which Cu_2_O nanowires are served as the sacrificial template, and Na_2_S_2_O_3_ and CoCl_2_ are used as coordination etching agent and cobalt precursor, respectively. The chemical reaction is described as^30^

(1) $${\mathrm{Cu}}_{2}O+xS_{2}O_{3}^{\mathrm{2–}}+H_{2}O\to {\left[{\mathrm{Cu}}_{2}{\left(S_{2}O_{3}\right)}_{x}\right]}^{2-2x}+2{\mathrm{OH}}^{–}$$

(2) $${\mathrm{Co}}^{2+}+2{\mathrm{OH}}^{-}\to \mathrm{Co}{\left(\mathrm{OH}\right)}_{2}$$

When Na_2_S_2_O_3_ is added into Cu_2_O suspension, S_2_O_3_^2−^ starts to coordinate and etch Cu_2_O by forming a soluble complex [Cu_2_(S_2_O_3_)*_x_*]^2–2*x*^, and OH^−^ ions are released from this etching process (Equation(1)). These OH^−^ ions accumulate near the original Cu_2_O nanowire surface and encounter Co^2+^, and thus Co(OH)_2_ is precipitated in a nanosheet morphology (Equation (2)), in analogy of the formation of Co_3_O_4_ nanosheets reported recently.^31^ As this process only takes place around the Cu_2_O etching interface where the local OH^−^ concentration is the highest, Co(OH)_2_ nanosheets are continuously grown over the surface of the original Cu_2_O nanowires, with a constrained nanosheet thicknesses and a high density. These ultrathin Co(OH)_2_ nanosheets are further self‐assembled into a nanotube morphology, in which the original Cu_2_O nanowire templates are etched and formed the hollow interior. These hierarchical CoO*_x_* nanosheet/nanotube structures are further entangled to form a highly porous, conducting framework. After dehydration in vacuum (samples designated as CoO*_x_*‐vacuum) or in air (samples designated as CoO*_x_*‐air), the hierarchical CoO*_x_* nanosheet/nanotube structures are obtained.

**Figure 2:**
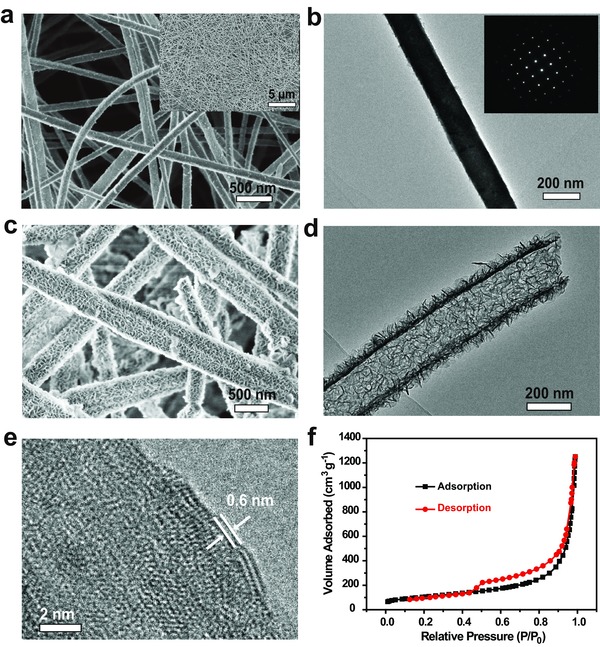
Structural characterization. a) SEM images, b) TEM image of the Cu_2_O nanowire templates. Inset of (b) is the SAED pattern of a single Cu_2_O nanowire. c) SEM, d) TEM, e) high‐resolution TEM images, and f) N_2_ adsorption–desorption isotherms of the CoO*_x_* nanosheet/nanotube heterostructure.

The as‐synthesized Cu_2_O nanowires are uniform, with an average diameter of 100–200 nm and a length of 20–50 μm (**Figure** **2**a). Transmission electron microscopy (TEM) images and selected‐area electron diffraction (SAED) patterns show that the Cu_2_O nanowires are single crystals (Figure 2b). X‐ray diffraction pattern of the Cu_2_O nanowire powder exhibits distinct diffraction peaks that are well indexed to the Cu_2_O phase (PDF No. 78‐2076), confirming that the as‐synthesized nanowires are high‐quality Cu_2_O (Figure S1, Supporting Information). The uniform nanowire morphology and high quality of Cu_2_O make it an excellent template to build hierarchical micronanostructures.

After the conversion of Cu_2_O nanowire templates in a mixed solution of Na_2_S_2_O_3_ and CoCl_2_, the color of the solution dispersion changes from yellow to green, and the product can be deposited into any arbitrary substrate to form a continuous film with a uniform surface morphology (Figure S2, Supporting Information). Scanning electron microscopy (SEM) images show that the exterior diameter of the obtained CoO*_x_* nanotubes is about 200 nm (Figure 2c), slightly larger than the original Cu_2_O nanowire templates. The length of the nanotubes is over 20 μm (Figure S3a, Supporting Information), similar to that of the nanowire templates. No nanowire morphology is observed in the obtained nanotubes (Figures 2d and S3a,b, Supporting Information), indicating the original Cu_2_O nanowires are all dissolved during the conversion. The energy dispersive X‐ray (EDX) spectroscopy pattern shows some Cu impurities are remaining in the CoO*_x_* nanotubes, with a Co to Cu atomic ratio of about 8:1 (Figure S4, Supporting Information). High‐resolution TEM images show that these nanotubes are constructed by thin nanosheets, with thickness less than 1 nm (Figure 2e). Furthermore, these CoO*_x_* nanotubes are overlapping with each other to form a 3D porous framework, which provides conducting channels for efficient electron transport. Meanwhile, the large pores between individual nanotubes also allow for a low CoO*_x_* density, effective ion transport and large surface area exposure for the catalytic water oxidation reaction.

N_2_ sorption isotherm of the hierarchical CoO*_x_* nanosheet/nanotube structure shows a distinct hysteresis loop, suggesting a typical mesoporous structure (Figure 2f). An ultrahigh Brunauer–Emmett–Teller (BET) specific surface area of 371 m^2^ g^−1^ is achieved. To our best knowledge, this is the largest reported BET specific surface area value for cobalt oxides.^11^, ^21^, ^22^, ^24^, ^26^, ^31^ This ultrahigh surface area can be attributed to the assembly of ultrathin CoO*_x_* nanosheets into nanotubes and subsequently the 3D porous framework, which can avoid the aggregation of nanosheets and effectively maximize the surface area.

**Figure 3:**
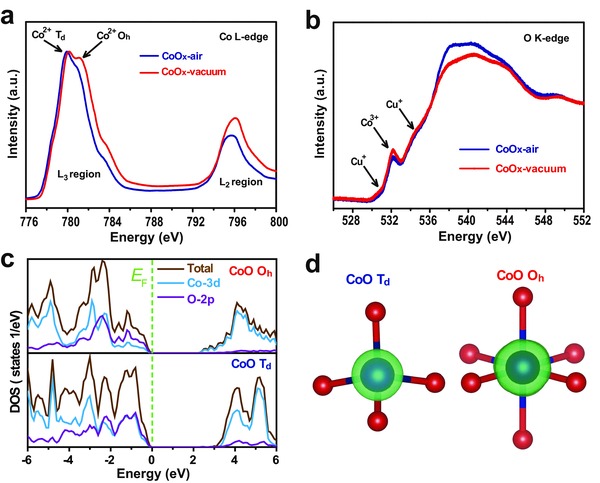
XAS spectra at a) Co L_3,2_ edge and b) O K‐edge of the CoO*_x_*‐vacuum (red curve) and CoO*_x_*‐air (blue curve) catalyst in TEY mode. The splitting of the L_3_ region at ≈780 eV in a) corresponds to the Co^2+^ T_d_ and Co^2+^ O_h_ species. c) The total DOS and the PDOS of CoO octahedral structure (O_h_) and tetrahedral structure (T_d_). d) Partial charge densities of the CoO T_d_ and O_h_ structures showing the more delocalization of electrons in the CoO O_h_ structure.

The electronic structure of CoO*_x_* is further characterized by X‐ray absorption spectroscopy (XAS), which is a synchrotron technique for studying the electronic and chemical structures of materials.^9^ The Co L‐edge XAS spectra of both CoO*_x_*‐air and CoO*_x_*‐vacuum samples were recorded in the total electron yield (TEY) mode (**Figure** **3**a), which can attain the surface sensitivity about 5 nm.^32^ Due to the orbital splitting, the Co L‐edge XAS splits into two regions, L_3_ and L_2_ regions centered at 780 and 796 eV, respectively.^33^ In L_3_ region, the peaks at 780 eV in both of the two CoO*_x_* samples are ascribed to Co^2+^ tetrahedral structure (designated as Co^2+^ T_d_).^34^ Noteworthy, the shoulder peak at 781.1 eV appears in the CoO*_x_*‐vacuum sample, which is ascribed to the crystal‐field redistribution by the Co^2+^ octahedral structure (designated as Co^2+^ O_h_).^34^ This unique Co^2+^ O_h_ structure can play an important role in the enhanced OER activity of the electrocatalyst.^16^, ^35^ The O K‐edge XAS was also recorded in TEY mode (Figure 3b). A prominent peak at 532 eV is observed in the preedge area of the CoO*_x_*‐air and CoO*_x_*‐vacuum samples, which is associated to the Co 3d‐O 2p interaction.^33^ This result suggests that both the CoO*_x_*‐air and CoO*_x_*‐vacuum samples have some Co^3+^ species. Two shoulder peaks are observed at 530.7 and 534 eV (Figure 3b), which are attributed to the Cu_2_O impurities in the CoO*_x_* samples, consistent with previous EDX result (Figure S4, Supporting Information).

Density functional theory (DFT) calculations are applied to compare the electronic structures of CoO‐O_h_ (rocksalt) and CoO‐T_d_ (zincblende) structures for water oxidation reaction, and both the total densities of states (DOS) and the projected densities of states (PDOS) of the O_h_ and T_d_ structures are plotted (Figure 3c). The valence band maximums of both structures are contributed mainly by Co 3d and a little of O 2p states. The calculated band gap energy for the Co^2+^ O_h_ structure is 2.4 eV, which is in good agreement with the experimental value^36^ and smaller than that of the Co^2+^ T_d_ structure (2.9 eV), suggesting that electrons in O_h_ structures are easier to be excited into the conduction band than that of T_d_ structures. In addition, the partial charge densities of the O_h_ structure is calculated, which exhibits a more delocalized profile than that of the T_d_ structure (Figure 3d), so the electrons around the Co^2+^ O_h_ structure are more active. Furthermore, the calculated work function (*W*_f_) of the Co^2+^ O_h_ structure is 0.46 eV smaller than that of Co^2+^ T_d_ structure, further indicating the higher capability of losing electrons from the O_h_ structure than the T_d_ structure. As the molecular mechanisms of water oxidation at Co sites involve reaction cycles among Co^2+^, Co^3+^, and Co^4+^ oxidation sites,^5^, ^16^ the Co^2+^ O_h_ structure with a smaller energy barrier can significantly facilitate the electron transfer process for water oxidation reaction. Our XAS experimental results have shown the existence of Co^2+^ O_h_, which are different from most of the previous reported OER catalysts with Co^2+^ T_d_ structures,^37^ thus providing an optimized electronic structure at the atomic scale for OER catalysis. In addition, the CoO*_x_*‐vacuum catalyst demonstrate even better OER activity than the CoO*_x_*‐air sample, which can be attributed to the higher Co^2+^ O_h_ concentration in the CoO*_x_*‐vacuum catalyst (as shown by the XAS data). These Co^2+^ O_h_ sites will transform into Co^2+^ T_d_ when the CoO­*_x_*‐vacuum samples are heated in oxygen, further supporting our hypothesis of optimizing the Co^2+^ O_h_ concentration.

**Figure 4:**
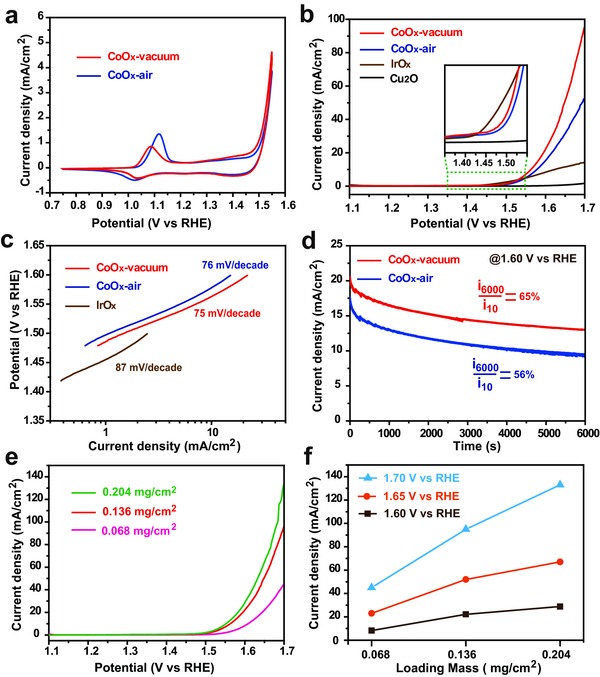
CoO*_x_* nanotubes as water oxidation electrocatalyst. a) CVs of the CoO*_x_*‐vacuum (red curve) and CoO*_x_*‐air (blue curve) catalysts at 5 mV s^−1^. b) Water oxidation current of the CoO*_x_*‐vacuum (red curve), CoO*_x_*‐air (blue curve), IrO*_x_* (brown curve), and Cu_2_O nanowire (black curve) at 5 mV s^−1^. Inset: The zoom‐in plot of the onset potential region. c) Tafel plots of water oxidation current in (b). d) Stability test at 1.60 V versus RHE. All the samples were loaded onto GC electrodes with the same loading mass of 0.136 mg cm^−2^ and tested in 1 m KOH. e) Water oxidation current of the CoO*_x_* nanotubes at different loading mass scanning at 5 mV s^−1^. f) Specific current densities at 1.60, 1.65, and 1.70 V versus RHE of the CoO*_x_*‐vacuum catalysts at different loading mass.

The electrochemical behaviors of CoO*_x_* samples (both dried in air and in vacuum) are first tested by cyclic voltammograms (CVs) between 0.75 and 1.55 V (vs RHE) (**Figure** **4**a). The double layer regions below 0.9 V for both catalysts are overlapping, suggesting an approximately similar surface area exposure from a constant catalyst loading.^38^ A major oxidation peak is observed at 1.0–1.2 V, corresponding to the conversion of Co^2+^ to Co^3+^.^21^, ^39^ In addition, this oxidation potential for the CoO*_x_*‐vacuum sample (red curve) is negatively shifted for 40 mV compared to that of the CoO*_x_*‐air sample (blue curve). When the potential reaches above 1.45 V, a sharp increase of oxidation current occurs for both samples, due to the water splitting.

The OER activity of the hierarchical CoO*_x_* nanosheet/nanotube structures (both dried in air and in vacuum) are then evaluated by linear sweep voltammetry (LSV) between 1.10 and 1.70 V versus RHE (Figure 4b). For comparison, the Cu_2_O nanowire precursor and IrO*_x_* nanoparticles with a same mass loading of 0.136 mg cm^−2^ are tested under similar conditions. The current density of pristine Cu_2_O nanowire (black curve) is much smaller than CoO*_x_*, suggesting a negligible electrochemical interference resulted from the remaining Cu_2_O impurity. The IrO*_x_* nanoparticle has the lowest onset potential of ≈1.41 V versus RHE (brown curve), while its current density at higher potentials is still much lower than both of the CoO*_x_* samples. The CoO*_x_*‐vacuum nanosheet/nanotube catalyst exhibits the best OER performance among all these samples. The current density at 1.65 V versus RHE reaches 51.2 mA cm^−2^, which is 1.6 times higher than that of the CoO*_x_*‐air catalyst (31.5 mA cm^−2^) and 4.5 times higher than that of IrO*_x_* nanoparticles (11.5 mA cm^−2^). The onset potentials of the CoO*_x_*‐vacuum and CoO*_x_*‐air catalysts are 1.46 and 1.48 V, respectively, which are substantial lower than almost all of the previously reported CoO*_x_* OER catalysts^9^, ^10^, ^11^, ^12^, ^18^, ^21^, ^23^, ^24^, ^26^, ^40^ (see summarized references in Table S1, Supporting Information).

To further compare the electrocatalytic water splitting activity, Tafel plots of the hierarchical CoO*_x_* nanosheet/nanotube structures are displayed, in comparison with the IrO*_x_* nanoparticles (Figure 4c). The Tafel slopes of the CoO*_x_*‐vacuum and the CoO*_x_*‐air catalysts are 75 and 76 mV dec^−1^, respectively, lower than that of the IrO*_x_* nanoparticles (87 mV dec^−1^), suggesting more efficient charge transfer on these nanotubes. Furthermore, the electrocatalytic stability of the CoO*_x_*‐vacuum and the CoO*_x_*‐air catalysts are evaluated by chronoamperometry measurement (Figure 4d). After 6000 s, the CoO*_x_*‐vacuum catalyst remains at a current density of 13.0 mA cm^−2^ at 1.60 V versus RHE, which is ≈65% of its initial activity, outperforming the values of 9.3 mA cm^−2^, and 56% of activity retention for the CoO*_x_*‐air catalyst.

To compare with other reported OER catalysts, the OER activity of the CoO*_x_*‐vacuum catalyst has been further tested in O_2_‐saturated 0.1 m KOH (Figure S5a, Supporting Information). The comparison of the LSV curves recorded in 0.1 m KOH and in 1 m KOH shows that the onset potentials are nearly identical, while the current density observed in 0.1 m KOH is ≈70% of that observed in 1 m KOH. This is probably attributed to a lower OH^−^ concentration in the electrolyte, which is consistent with previous reports.^26^ Additional cyclic voltammetry sweeps show that the current density of the catalyst decreases to ≈70% after 200 cycles of continuous sweeping (Figure S5b, Supporting Information), consistent with the chronoamperometry measurement shown in Figure 4d.

Moreover, the mass loading effect of the CoO*_x_*‐vacuum catalyst is further investigated to demonstrate the efficient mass transport within such a hierarchical structure. The LSV curves of the CoO*_x_*‐vacuum catalyst, with different mass loading of 0.068, 0.136 and 0.204 mg cm^−2^, are displayed (Figure 4e), and the current densities at three selected potentials of 1.60, 1.65, and 1.70 V versus RHE are summarized (Figure 4f). It can be seen that the current density increases almost linearly with the catalyst mass loading, benefiting from the large porous structure and homogeneous catalyst layer coating on the CoO*_x_* nanosheet/nanotube structure. To the best of our knowledge, the superior OER performance of the CoO*_x_*‐vacuum catalyst, including the low onset potential, high electrocatalytic current density, and stable catalytic activity, is the best compared to the state‐of‐the‐art Co‐based OER catalysts (Table S1, Supporting Information).

The much enhanced OER catalytic activity of the hierarchical CoO*_x_* nanosheet/nanotube framework can be attributed to its unique atomic and nanoscale structures, as well as the 3D assembled architecture. First, at the atomic scale, the Co^2+^ ions located at octahedral sites (Co^2+^ O_h_) exhibit better OER activity than Co^2+^ at tetrahedral sites (Co^2+^ T_d_), as demonstrated by the DFT calculations. Second, the CoO*_x_* nanosheet/nanotube heterostructure offers a unique building block for simultaneous optimization of high surface area and efficient 1D charge transport behavior. Distinct from the conventional, direct synthesis of CoO*_x_* from Co^2+^ and OH^−^ precursors, the use of Cu_2_O nanowire sacrificial templates allows for controlled, slow release of OH^−^ anions close to the surface of Cu_2_O, thus resulting in the replacement/growth of Co(OH)_2_ on the template surface and avoiding the fast homogeneous nucleation of Co(OH)_2_ in the solution. Importantly, the thin diameter of Cu_2_O nanowire templates is only sufficient to sustain the replacement reaction under a limited level in different positions of the nanowire surface, resulting in the ultrathin thickness (≈1 nm) of the grown Co(OH)_2_ nanosheets. Consequently, an ultrahigh surface area of 371 m^2^ g^−1^ is obtained, substantially larger than previously reported CoO*_x_* nanoparticles (111 m^2^ g^−1^),^41^ nanowires (58 m^2^ g^−1^),^21^ nanosheets (152 m^2^ g^−1^)^11^ and mesoporous nanostructures (156 m^2^ g^−1^).^26^ Moreover, the simultaneous etching of the Cu_2_O nanowire template leads to the formation of the nanotube structure that are further interlaced into a 3D framework, which not only favors the 1D electron transport over the nanosheet/nanotube heterostructure surface, but also facilitates the electrolyte diffusion inside the hollow nanotubes as well as between adjacent nanotubes.

**Figure 5:**
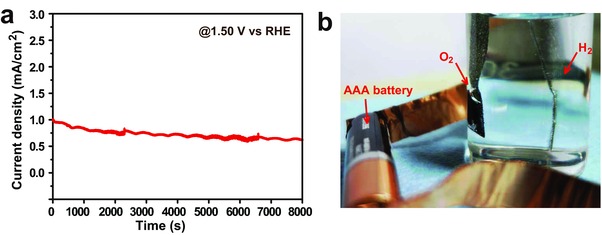
a) *I*–*t* test of the CoO*_x_* nanosheet/nanotube heterostructure catalyst at 1.50 V versus RHE. The CoO*_x_* catalyst was loaded onto GC electrodes with a loading mass of 0.136 mg cm^−2^ and tested in 1 m KOH. b) Demonstration of water splitting cell powered by a 1.5‐V AAA battery. The CoO*_x_* catalyst was loaded on Ni foam with a loading mass of 4 mg cm^−2^ as an anode and a Pt‐mesh was used as a cathode.

Due to its ultralow water oxidation overpotential, the hierarchical CoO*_x_* nanosheet/nanotube catalyst can produce ≈1 mA cm^−2^ current density at 1.50 V versus RHE, which remains 65% after 8000 s stability test (**Figure** **5**a). The capability of the hierarchical CoO*_x_* nanosheet/nanotube catalyst has further been demonstrated by a single‐battery water splitting experiment, in which the CoO*_x_* catalyst and a Pt mesh are used as the anode and cathode, respectively. When a single‐cell AAA battery with a voltage of 1.5 V is used as the energy source, continuous bubbles are observed from both the cathode and anode, suggesting successful water splitting (Figure 5b). This experiment suggests the potential of using the CoO*_x_* catalyst for efficient electrical water splitting in the industry scale in the future.

## 3 Conclusion

Inspired by plant leaves with optimized surface area and efficient mass transport, we have developed a CoO*_x_* nanosheet/nanotube heterostructure, by in situ etching of Cu_2_O nanowire template and regrowth of ultrathin CoO*_x_* nanosheets on the template surface. This unique 2D/1D architecture offers structural optimization over three different length scales. At the atomic scale, the formation of Co^2+^ octahedral sites leads to a smaller energy barrier that significantly facilitates the electron transfer process for water oxidation reaction. At the nanoscale, the two‐step growth method provides an effective means of limiting the homogeneous nucleation of CoO*_x_* nanosheets in solution and over‐accumulation of nanosheet thickness, thus resulting in an ultrahigh surface area of 371 m^2^ g^−1^ and large exposure of active sites for the interfacial OER. Finally, at the microscale, the assembly of individual nanosheet/nanotube building blocks into a 3D, highly porous and electrically conducting framework allow for efficient transport of charges and electrolytes, thus significantly contributing to the OER catalytic activity. As a result, the CoO*_x_*‐vacuum catalyst shows a low onset potential of ≈1.46 V versus RHE that is comparable to IrO_2_ catalyst, and a record‐high OER current density of 51.2 mA cm^−2^ at 1.65 V versus RHE and a Tafel slope of 75 mV dec^−1^. Finally, a full water splitting cell with the CoO*_x_* nanosheet/nanotube catalyst and a Pt‐mesh is demonstrated, under a single 1.5‐V battery as the power supply. Our study demonstrates an effective way to realize a novel 3D architecture with multiscale optimization, which will open up a variety of opportunities of other metal chalcogenides^42^, ^43^, ^44^ for energy applications.

## 4 Experimental Section

*Material Synthesis*: The Cu_2_O nanowires were synthesized by Fehling's test reaction. Typically, an 800 mL of solution containing 0.625 × 10^−3^ m CuSO_4_, 4.375 × 10^−3^ m NaOH, 2.5 × 10^−3^ m sodium tartrate, and 0.125 × 10^−3^ m glucose was mixed as the precursor, and sealed in a container and heated at 95 °C for 90 min. After the reaction, the yellow product was collected by centrifugation, washed by deionized (DI) water and ethanol repeatedly for three times, and dried in a 45 °C oven to obtain the Cu_2_O nanowires. The hierarchical CoO*_x_* nanosheet/nanotube structures were synthesized by using a coordinating etching and precipitating method. Typically, 200 mg of Cu_2_O nanowires and 68 mg of CoCl_2_·6H_2_O were dispersed in 200 mL of water and 200 mL of ethanol to form a suspension. Then 160 mL of Na_2_S_2_O_3_ (1 m) was added into the suspension dropwise. The color of the Cu_2_O solution is obscure orange. After excessive amount of Na_2_S_2_O_3_ was added into the solution, the color of the solution turned from light orange to transparent green at the initial 5 min, and we kept stirring for 30 min to convert most of Cu_2_O into Co(OH)_2_. The product was collected by centrifugation, washed by DI water and ethanol repeatedly for three times and dried at 80 °C in vacuum or air to get the hierarchical CoO*_x_* nanosheet/nanotube structures. The IrO*_x_* nanoparticle catalysts were synthesized as follows. A 300 mL of sodium hexachloroiridate(IV) hexahydrate solution (1 × 10^−3^ m) was mixed with 3 mL of potassium hydroxide (0.1 m) and heated in 90 °C for 6 h. The products were collected by centrifugation, washed with DI water and ethanol repeatedly for three times, and then dried in the air.

*DFT Calculations*: All calculations reported in this work were performed by using the Vienna ab initio simulation package (VASP)^45^ in the framework of the projector augmented wave method with a cutoff for the plane waves of 550 eV.^46^ A Hubbard‐like, localized term was added to the Perdew‐Burke‐Ernzerhof (PBE) generalized gradient approximation (GGA) exchange correlation functional, called GGA+U. The *U*_eff_ (*U* −*J*) values of 6 eV were applied for Co 3d states. Both rocksalt CoO and zincblende CoO are used 2 × 2 × 2 super cells, with 8 × 8 × 8 mesh of k‐points. All atoms were fully relaxed with the energy convergence tolerance of 10^−6^ eV atom^−1^ and the final force on each atom was <0.01 eV Å^−1^. The work function is defined as the energy of the vacuum level with respect to Fermi level of the system, which is calculated from VASP. Both rocksalt CoO(111) and zincblende CoO(111) surfaces were modeled using symmetric slabs with vacuum widths of 15 Å for work function calculations.

*OER Catalytic Measurement*: The electrochemical measurements were performed with a CHI 660D electrochemistry workstation (CHI Instrument Inc, USA). The electrolyte was 1 m KOH, which was deaerated with high‐purity Ar prior to and throughout all the measurements. The working electrode was a thin layer of Nafion‐impregnated catalyst cast on a glassy carbon rotating disk electrode (GC–RDE, 5 mm diameter, 0.196 cm^2^). The catalyst solution was prepared by mixing 1 mL of C_2_H_5_OH, 2 mg of catalyst and 120 μL of Nafion, and then pipetted onto a freshly polished GC–RDE to achieve the desired catalyst loading (e.g., 0.068, 0.136, and 0.204 mg cm^−2^). A Pt gauze and a saturated calomel electrode electrode were used as the counter and reference electrodes, respectively. The GC–RDE was controlled at 1000 rpm by a speed regulator (Pine Instrument, USA) to exclude the interference of generated oxygen bubbles at electrode surface. The scan rate for CV and LSV was kept at 5 mV s^−1^. All the electrochemical measurements were iR‐compensated and carried out at 25 ± 1 °C. The electrochemical tests in Figures S5 and S6, Supporting Information were tested in 0.1 m KOH presaturated by O_2_. The internal resistance *R*_s_ was determined from Nyquist plot and all the electrochemical measurements were *iR*‐compensated at ≈80% of the *R*_s_ and carried out at 25 ± 1 °C.

## Supporting information

As a service to our authors and readers, this journal provides supporting information supplied by the authors. Such materials are peer reviewed and may be re‐organized for online delivery, but are not copy‐edited or typeset. Technical support issues arising from supporting information (other than missing files) should be addressed to the authors.

Supplementary
