# Supplementary material for: Bio‐Inspired Leaf‐Mimicking Nanosheet/Nanotube Heterostructure as a Highly Efficient Oxygen Evolution Catalyst
Source: Adv Sci (Weinh). 2015 Mar 10;2(4):1500003. doi: 10.1002/advs.201500003 (PMC5024083; doi:10.1002/advs.201500003)
Supplement: Supplementary file 1 — Supplementary [file ADVS-2-0i-s001.pdf]

Copyright WILEY-VCH Verlag GmbH & Co. KGaA, 69469 Weinheim, Germany, 2015.

ADVANCED SCIENCE
Open Access

Supporting Information

for *Adv. Sci.*, DOI: 10.1002/advs.201500003

Bio-Inspired Leaf-Mimicking Nanosheet/Nanotube Heterostructure as a Highly Efficient Oxygen Evolution Catalyst

Yongcheng Wang, Kun Jiang, Hui Zhang, Tong Zhou, Jiwei Wang, Wei Wei, Zhongqin Yang, Xuhui Sun, Wen-Bin Cai,* and Gengfeng Zheng*

Copyright WILEY-VCH Verlag GmbH & Co. KGaA, 69469 Weinheim, Germany, 2013.

Supporting Information

Bio-inspired Leaf-mimicking Nanosheet/Nanotube Heterostructure as Highly Efficient Oxygen Evolution Catalyst

Yongcheng Wang, Kun Jiang, Hui Zhang, Tong Zhou, Jiwei Wang, Wei Wei, Zhongqin Yang, Xuhui Sun, Wen-Bin Cai, and Gengfeng Zheng**

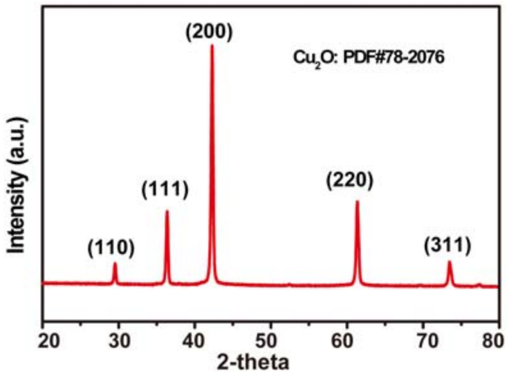

Fig. S1. XRD pattern of the Cu_2O nanowires.

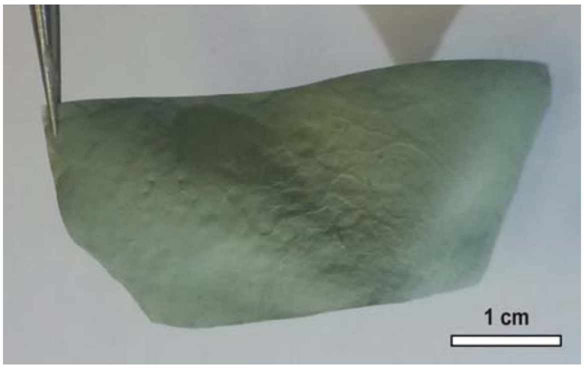

Figure S2. Photograph of the 3D CoO_x nanosheet/nanotube heterostructure framework deposited on a piece of paper.

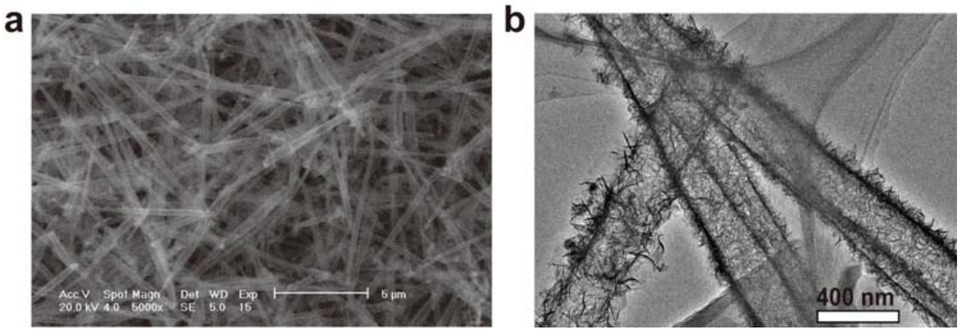

Figure S3. (a) SEM image and (b) TEM image of the CoO_x nanosheet/nanotube heterostructure.

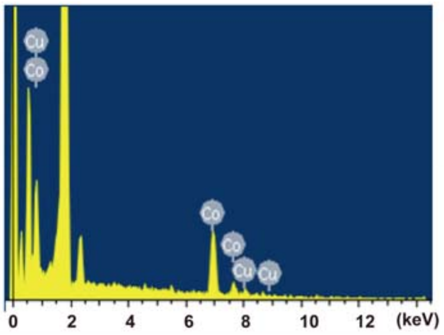

Element	Weight (%)	Atom (%)
Co K	87.97	88.75
Cu K	12.03	11.25
Total	100.00	

Figure S4. EDX pattern of the CoO_x nanosheet/nanotube heterostructure.

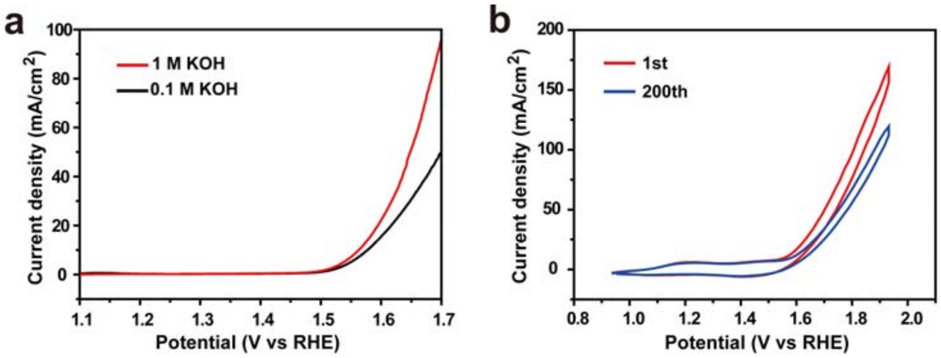

Figure S5. (a) linear sweep voltammetry (LSV) of the CoO_x-vacuum catalyst tested in O_2-saturated 0.1 M KOH (black curve). The catalyst tested in 1 M KOH as showed before is for comparison (red curve). (b) Continuous cyclic voltammetry curves sweeps of the CoO_x-vacuum catalyst at 100 $mV \cdot s^{-1}$ in 0.1 M KOH.

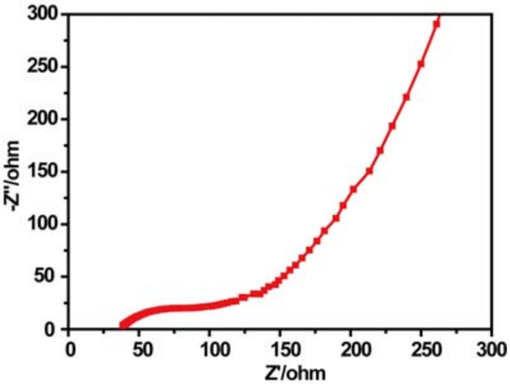

Figure S6. Nyquist plot of the CoO_x-vacuum catalyst tested in O_2-saturated 0.1 M KOH.

Table S1. Comparing of OER performance of this work with other literatures

Electrocatalysts	Mass loading (mg cm^{-2})	Onset potential (V vs. RHE)	Current density at 1.65 V (mA cm^{-2})
Hierarchical CoO_x nanosheet/nanotube	0.136	1.46	52
Electrochemically delithiated $LiCoO_2$ [1]	0.1	1.52	<15
Spinel-type delithiated $LiCoO_2$ [2]	0.25	1.56	15
Co_3O_4-Carbon Porous Nanowire Arrays [3]	0.2	1.47	60
Au@Co_3O_4 core shell [4]	0.064	1.52	22
Reduced mesoporous Co_3O_4 NWs [5]	0.136	1.52	13.1
Atomically-thin porous Co_3O_4 sheets [6]	0.34	1.56	50
N-rGO/$CoSe_2$ composite [7]	0.2	1.52	40
Photochemical deposition, amophous CoO_x [8]	--	1.44	7
Mesoporous Co_3O_4 [9]	0.13	1.60	4
Co_3O_4 NPs/N-rmGO composite[10]	0.24	1.50	20

References

1. Z. Lu, H. Wang, D. Kong, K. Yan, P. C. Hsu, G. Zheng, H. Yao, Z. Liang, X. Sun, Y. Cui, *Nat. Commun.* **2014**, *5*, 4345.
2. T. Maiyalagan, K. A. Jarvis, S. Therese, P. J. Ferreira, A. Manthiram, *Nat. Commun.* **2014**, 3949.
3. T. Y. Ma, S. Dai, M. Jaroniec, S. Z. Qiao, *J. Am. Chem. Soc.* **2014**, *136*, 13925.
4. Z. Zhuang, W. Sheng, Y. Yan, *Adv. Mater.* **2014**, *26*, 3950.

5. Y. Wang, T. Zhou, K. Jiang, P. Da, Z. Peng, J. Tang, B. Kong, W. B. Cai, Z. Yang, G. Zheng, *Adv. Energy Mater.* **2014**, DOI: 10.1002/aenm.201400696

6. Y. Sun, S. Gao, F. Lei, J. Liu, L. Liang, Y. Xie, *Chem. Sci.* **2014**, *5*, 3976.

7. M. R. Gao, X. Cao, Q. Gao, Y. F. Xu, Y. R. Zheng, J. Jiang, S. H. Yu, *ACS Nano* **2014**, *8*, 3970.

8. R. D. Smith, M. S. Prévot, R. D. Fagan, Z. Zhang, P. A. Sedach, M. K. J. Siu, S. Trudel, C. P. Berlinguette, *Science* **2013**, *340*, 60.

9. H. Tüysüz, Y. J. Hwang, S. B. Khan, A. M. Asiri, P. Yang, *Nano Res.* **2013**, *6*, 47.

10. Y. Liang, Y. Li, H. Wang, J. Zhou, J. Wang, T. Regier, H. Dai, *Nat. Mater.* **2011**, *10*, 780.
